# Cellular context and ligand class shape CXCR4-CCR5 heteromerization in live cells

**DOI:** 10.21203/rs.3.rs-9499667/v1

**Published:** 2026-05-20

**Authors:** Mohamed Seghiri, Mojeed Ashiru, Chiako Farshadfar, Elizabeth Hair, Andres Ruiz, Ceyvion Wilkins, Adam W. Smith

**Affiliations:** Texas Tech University, Department of Chemistry & Biochemistry, Lubbock, TX USA

## Abstract

G protein-coupled receptors (GPCRs) can form heteromeric assemblies, yet whether class A GPCR heteromerization is dictated by cellular context remains unclear. Here we identify CXCR4-CCR5 heteromerization as a cell-dependent, cholesterol-sensitive, and ligand-regulated feature of live-cell membrane organization. In cancer-derived MDA-MB-231 cells, CXCR4 and CCR5 formed stable, slow-diffusing higher-order assemblies, whereas in COS7, HEK293, and MCF-10A cells the receptors were detected mainly as weaker monomer-dimer mixtures. Cholesterol depletion selectively reduced CXCR4-CCR5 heteromerization in MDA-MB-231 cells, implicating membrane composition as a major determinant of receptor assembly. Agonists induced transient heteromerization coupled to receptor internalization, while antagonists, especially plerixafor together with maraviroc, stabilized persistent surface-associated complexes. Molecular dynamics simulations in asymmetric bilayers resembling MDA-MB-231 and MCF-10A membranes identified cholesterol-enriched receptor interfaces that prolong CXCR4-CCR5 dimer lifetimes in MDA-like membranes. These results show that GPCR heteromerization is not an intrinsic fixed property of receptor pairs, but an emergent behavior shaped by cell state, lipid environment, and ligand input.

## Introduction

Despite growing evidence that G protein-coupled receptors (GPCRs) can form oligomeric assemblies, the extent and physiological role of GPCR heteromerization remains a subject of considerable debate^1–5^. While class C GPCRs exhibit well-defined, constitutive dimerization, class A GPCRs form weaker, more transient interactions that are often modulated by receptor heteromerization, ligand binding, and cellular context^6^. Moreover, the dynamic and low-affinity nature of class A GPCR heteromers has made them notoriously difficult to detect with precision in living systems^7^. Importantly, no studies to date have directly assessed whether the heteromerization of class A GPCRs is modulated by cell type or disease state under physiological conditions.

A central limitation in the field lies in the available methods that can resolve GPCR heteromers with both spatial and temporal resolution at native abundance^7^. Förster resonance energy transfer (FRET) and bioluminescence resonance energy transfer (BRET) have provided early evidence for GPCR proximity, but they don’t directly measure receptor surface density, diffusion coefficients, or oligomeric stoichiometry, parameters that are essential for defining the composition and function of GPCR assemblies in situ^8^. Moreover, because these assays often require receptor overexpression above physiological levels, they are prone to apparent positives arising from stochastic co-localization and fleeting collisions^9^. Single molecule techniques offer high spatial resolution, yet they demand sparse labeling and very-low expression levels (≤1 molecule $\mu {\text{m}}^{-2}$), below the abundance of many GPCRs in cancer cells^10^. They are further limited by photophysics, labeling chemistry, and low throughput^11–13^. By contrast, fluorescence correlation spectroscopy (FCS/FCCS) techniques provide the needed resolution to quantitatively measure density, mobility, and oligomeric state^14,15^. A time-correlated single-photon implementation of FCCS called pulsed interleaved excitation-FCCS (PIE-FCCS) removes spectral bleed-through, enabling accurate cross-correlation of overlapping fluorophore emissions^16^. PIE-FCCS quantifies receptor mobility and dimerization at physiologically relevant surface densities, with sensitivity from ~10 to 1,000 molecules $\mu {\text{m}}^{-2}$^17^. From a single acquisition, it returns multiple parameters including oligomerization, diffusion coefficients, molecular brightness (stoichiometry/complex size), and receptor number density, enabling real-time detection of weak, transient heteromers at native expression levels with statistical power across hundreds of cells.

Direct high-resolution structure determination of class A GPCR heteromers also remains challenging due to their dynamic interfaces, large complex size, and sensitivity to the membrane environment^7^. As an alternative, molecular dynamics (MD) simulations are a powerful approach to model the conformational landscape and free-energy profiles of GPCR heterodimers, revealing cholesterol-sensitive dimer interfaces and binding modes that would otherwise remain experimentally elusive^18^. Thus, computational modeling and biophysical live-cell methods such as PIE-FCCS remain critical for defining the functional architecture of GPCR heteromers under near-native conditions.

The chemokine receptors CXCR4 and CCR5 provide an interesting model system for interrogating class A GPCR heteromerization in live cells. They are critical mediators of immune cell trafficking, inflammation, and cancer metastasis^19^. Individually, these receptors have been extensively studied for their roles in HIV-1 entry and tumor progression^20,21^. However, growing evidence suggests that they do not function solely as monomers but can form dynamic complexes that modulate ligand recognition, signal transduction, and receptor trafficking^18,22–30^. Notably, the physical and functional interaction between CXCR4 and CCR5 has emerged as a potentially important mode of GPCR regulation, though it remains poorly characterized in live cellular contexts.

In this study, we used PIE-FCCS to quantify CXCR4-CCR5 heteromerization in live cells, comparing normal (COS7, HEK293, MCF-10A) and cancer-derived (MDA-MB-231) cell lines to test modulation by cellular context and membrane composition. Our results demonstrate a striking cell type-dependent bias in CXCR4-CCR5 heteromerization, with significantly enhanced interactions observed in MDA-MB-231 breast cancer cells compared to other lines under basal conditions. Ligands further reshape this CXCR4-CCR5 heteromer landscape. Antagonists, particularly the plerixafor and maraviroc combination, stabilize the CXCR4-CCR5 heteromerization in both MDA-MB-231 and MCF-10A, yielding sustained cross-correlation and slower apparent diffusion. In contrast, agonists (CXCL12, CCL5) induce a transient heteromerization that decays over time, consistent with ligand-driven internalization and loss of co-diffusing complexes from the plasma membrane. These findings support the existence of context-specific receptor crosstalk and suggest that CXCR4-CCR5 heteromers may contribute to aberrant GPCR signaling in cancer. By establishing PIE-FCCS as a powerful approach to monitor receptor heteromerization in live cells, this work provides mechanistic insight into GPCR organization and opens new avenues for targeting GPCR heteromers in disease.

## Results

### Experimental benchmarking of heterodimerization measured by PIE-FCCS on live-cell plasma membrane

**Figure 1.**
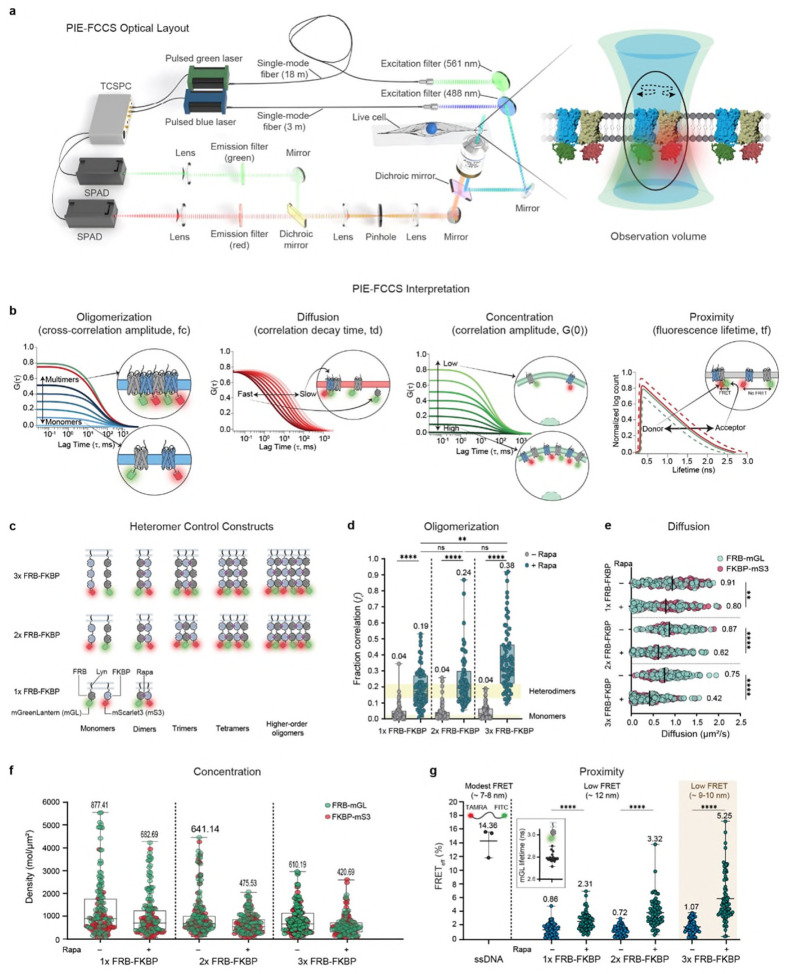
Validation of heteromers detection with FRB-FKBP controls in COS-7 cells. **a**, Schematic of the PIE-FCCS optical setup showing the pulsed 488 nm and 561 nm excitation lasers and the optical paths of excitation and emission light through the various microscope components to the single-photon avalanche detectors (SPADs) and the time-correlated single-photon counting (TCSPC) system, and the position of the observation volume. The observation volume and membrane proteins are not drawn to scale; the radius of the observation volume is approximately 200-fold larger than the size of the membrane proteins. **b**, Schematic overview of the multiparameter readouts obtained from a single PIE-FCCS measurement, including oligomerization, diffusion (mobility), concentration (density), and intermolecular proximity assessed by FRET. **c**, Cartoon of expected FRB-FKBP assembly states (1x, 2x, 3x). **d**, Fractional cross-correlation ($f_{c}$) reporting heterodimer state before and after adding rapamycin (heterodimerization inducer). **e**, Diffusion coefficients of FRB-FKBP species before and after adding rapamycin. **f**, Surface number density (molecules per ${\mu \text{m}}^{2}$) of FRB-FKBP constructs before and after adding rapamycin at 100 nM final. **g**, FRET efficiency of FRB-FKBP constructs before and after adding rapamycin and for a 40-nt ssDNA standard labeled with 5’ TARMA and 3’ FITC; inset, mGreenLantern fluorescence lifetime from the 1xFKBP-FRB-mGL construct. Box-and-whisker plots (**d**, **f**): boxes denote the 25^th^-75^th^ percentiles, center lines the median, whiskers the range, and the value above each plot is the mean. Lines in **e** and **g** indicate the median and the numbers indicate the mean. Data was analyzed using Kruskal-Wallis tests, followed by Dunn’s multiple-comparisons correction; **** and ** denote P < 0.0001 and P < 0.01, respectively. Data was combined from 3 independent days; on each day ~30–40 cells were measured before and after ligand addition.

We first established a PIE-FCCS workflow that yields benchmarked readouts of receptor organization at the plasma membrane (Fig. 1a). A pulsed supercontinuum source with interleaved 488/561-nm excitation and time-gated detection suppresses spectral bleed-through and afterpulsing, enabling accurate calculation of dual-color auto- and cross-correlation functions (ACF/CCF). The confocal detection geometry was quantified with a dual-labeled 40-nt DNA standard (3D diffusion) to co-align excitation and detection volumes in x-y (Supplementary Fig. 1a), and with supported lipid bilayers (SLBs; 2D diffusion), to verify axial co-alignment while scanning the dual excitation and detection volumes along z axis (Supplementary Fig. 1c,d). These calibrations define the effective observation area/volume and, together with model fits to ACF/CCF, provide, within the same acquisition, fractional cross-correlation (homomerization/heteromerization), diffusion coefficients, surface density, and proximity (Fig. 1b). Day-to-day reproducibility of the CCF amplitude was tracked with the DNA standard (Supplementary Fig. 1b), and axial co-registration was confirmed by z-scanning SLBs labeled with spectrally distinct lipids (Supplementary Fig. 1e,f).

To demonstrate multiparameter performance in cells, we designed rapamycin-inducible FRB-FKBP assemblies targeted to the plasma membrane of COS7 cells via a Lyn anchor, with FRB and FKBP fused to mGreenLantern (mGL) and mScarlet3 (mS3), respectively, and repeated in tandem (1x, 2x, 3x) to enforce higher valency (Fig. 1c; Supplementary Fig. 2a,b). Prior to induction, fractional cross-correlation ($f_{c}$) values were low, 0.042 ± 0.156 (1x), 0.045 ± 0.055 (2x), and 0.047 ± 0.045 (3x), consistent with predominantly monomeric species. Rapamycin (100 nM) increased $f_{c}$ in a valency-dependent manner to 0.194 ± 0.177 (1x), 0.242 ± 0.177 (2x), and 0.376 ± 0.212 (3x) (Fig. 1d). This scaling is consistent with prior chemically induced FKBP homodimerization benchmarks (AP20187; reported fc≈0.11/0.16/0.28 for 1x/2x/3x)^31^. Absolute fc values can shift with fluorophore pair, expression balance and construct architecture, which influence brightness, dark fractions and the effective correlated population detected by PIE-FCCS. Rapamycin-induced assembly was accompanied by reduced apparent diffusion, from 0.91 ± 1.81 to $0.80\pm 1.92\;\mu {\text{m}}^{2}\;{\text{s}}^{-1}$ (1x), 0.87 ± 1.68 to $0.62\pm 1.71\;\mu {\text{m}}^{2}\;{\text{s}}^{-1}$ (2x), and 0.75 ± 1.91 to $0.42\pm 1.63\;\mu {\text{m}}^{2}\;{\text{s}}^{-1}$ (3x) (Fig. 1e), consistent with formation of larger complexes. Measurements were obtained at stable surface densities (≈420.7–877.4 molecules $\mu {\text{m}}^{-2}$; Fig. 1f), within typical transient-expression ranges for membrane receptors in mammalian cells ($\approx 10-1000\;\mu {\text{m}}^{-2}$). Plotting $f_{c}$ versus surface density revealed a positive trend indicative of density-dependent increases in co-diffusion (Supplementary Fig. 2c).

As an orthogonal proximity control, we quantified FRET alongside PIE-FCCS (Fig. 1g). A dual-labeled 40-nt ssDNA (single-stranded DNA) reference exhibited modest FRET (14.36%), consistent with the expected 7–8 nm root-mean-square end-to-end distance of a flexible single strand (hairpin formation can make it much shorter) which is a distance that is outside a strong FRET range (R_0_ ≈ 5–6 nm)^32^. FRB-FKBP constructs displayed low absolute FRET (0.86–1.07%), consistent with the limited spectral overlap of the mGL-mS3 pair, but increased upon rapamycin and with higher valency (2.31–5.25%), mirroring the rise in $f_{c}$. While FRET alone cannot specify oligomeric state due to distance/orientation constraints, PIE-FCCS reports co-diffusion independent of chromophore geometry. Together, the FRB-FKBP data and our calibration workflow establish that this PIE-FCCS approach delivers robust, multi-parameter characterization of oligomerization, diffusion, density, and proximity in living cells.

### CXCR4-CCR5 exhibits cell-dependent heteromerization

We next performed a literature-guided comparison of chemokine-receptor pairings in COS7 cells to contextualize CXCR4-CCR5 heteromerization against other previously reported heteromers^33,34^ (Supplementary Fig. 3). Under matched expression windows, PIE-FCCS revealed a clear hierarchy. CXCR4-CCR3 showed near-baseline fractional cross-correlation ($f_{c}$) at 0.03 ± 0.07 and fast diffusion at $0.39\pm 0.71\;\mu {\text{m}}^{2}\;{\text{s}}^{-1}$ consistent with predominantly monomeric behavior. CXCR4-CCR5 displayed intermediate $f_{c}$ (0.09 ± 0.17) accompanied by slower diffusion ($0.24\pm 0.66\;\mu {\text{m}}^{2}\;{\text{s}}^{-1}$) indicative of some dimer formation. In contrast, CXCR4-CXCR7 exhibited the strongest co-diffusion signature $f_{c}$ (0.20 ± 0.17) with the slowest apparent mobility ($0.27\pm 0.59\;\mu {\text{m}}^{2}\;{\text{s}}^{-1}$), consistent with high affinity dimerization and perhaps higher-order oligomers (Supplementary Fig. 3a,b). As a reference, we previously measured CXCR4 homomerization by PIE-FCCS in COS7 cells, yielding a mean $f_{c}$ of 0.18 ± 0.17, consistent with simple dimerization^31^. To anchor these measurements to experimental standards, we compared each receptor pairing to the 1x FRB-FKBP baseline before rapamycin (negative control; $f_{c}\approx 0.04$) and the enforced 1x FRB-FKBP heterodimer after rapamycin (positive reference; $f_{c}\approx 0.19$). Relative to these controls, CXCR4-CCR3 remained indistinguishable from baseline (ns), CXCR4-CCR5 increased modestly above baseline (*) but remained significantly below the enforced heterodimer (****), and CXCR4-CXCR7 approached the heterodimer reference without a detectable difference (ns) (Supplementary Fig. 3a). Representative ACF/CCF traces mirrored these trends, with cross-correlation amplitude scaling from CCR3 (lowest) to CCR5 (modest) to CXCR7 (highest) at comparable expression levels (Supplementary Fig. 3c).

To assess the influence of cellular context, we generated MDA-MB-231 CXCR4 knockout (CXCR4-KO) cells using CRISPR-Cas9 (Supplementary Fig. 4a). Loss of surface CXCR4 was confirmed by flow cytometry using APC-anti-CXCR4 staining with isotype controls (Supplementary Fig. 4b). Unless otherwise noted as wild type (WT), subsequent mentions of MDA-MB-231 refer to the CXCR4-KO line. To control for the influence of each cell line on diffusion and cross-correlation, we measured mGluR2 (a covalent dimer control) and Δ2Δ (monomer control) across COS7, HEK293, MCF-10A, and MDA-MB-231. In each background, mGluR2 consistently yielded high $f_{c}$ (0.14 ± 0.05 – 0.17 ± 0.24) with slower diffusion ($0.15\pm 0.33-0.27\pm 0.48\;\mu {\text{m}}^{2}\;{\text{s}}^{-1}$), whereas Δ2Δ remained near baseline with low $f_{c}$ (0.01 ± 0.09 – 0.03 ± 0.10) and faster diffusion ($0.40\pm 1.56-0.80\pm 1.11\;\mu {\text{m}}^{2}\;{\text{s}}^{-1}$) (Supplementary Fig. 4c-e). When analyzed across cell lines for each construct separately, fc distributions were indistinguishable, whereas diffusion coefficients differed significantly between cell backgrounds (Kruskal-Wallis with Dunn’s multiple comparisons; Supplementary Table 6) consistent with cell-specific membrane and cortical constraints on lateral mobility. Together, these controls validate the specificity of fc-based oligomerization assignments across cell types while highlighting that absolute diffusion is inherently context dependent.

**Figure 2.**
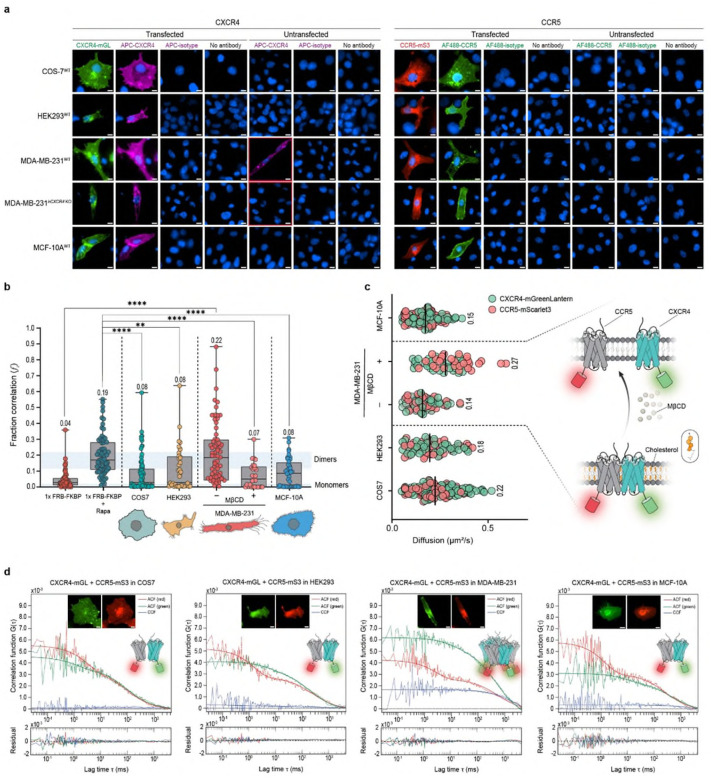
CXCR4 and CCR5 expression and heteromerization across mammalian cell lines. **a**, Immunofluorescence of endogenous and overexpressed receptors in WT COS7, HEK293, MDA-MB-231, and MCF-10A, and CXCR4-KO MDA-MB-231. Cells were plated in 96 well plates, transfected with CXCR4-mGL (0.4μg/well), fixed (4% paraformaldehyde), stained with APC-conjugated anti-human CXCR4 (5μL of 100μg/mL) or APC-conjugated mouse IgG2a, k isotype control (5μL of 100μg/mL), AF488-conjugated anti-human CCR5 (10μL of 100μg/mL) or APC-conjugated rat IgG2b, k isotype control (10μL of 100μg/mL), and counterstained with DAPI (0.8μg/mL). Staining was initiated 24 h after transfection, and imaging was performed immediately thereafter. **b**, Fractional cross-correlation ($f_{c}$) reporting CXCR4-CCR5 oligomerization in COS7, HEK293, MDA-MB-231 (CXCR4-KO), and MCF-10A. MDA-MB-231 shows elevated heteromerization that decreases upon methyl-β-cyclodextrin (MβCD) treatment at 5 mM final. Box-and-whisker plots show the 25^th^-75^th^ percentiles (box), median (center line), and range (whiskers), and the value above each plot is the mean. **c**, Apparent diffusion coefficients for CXCR4-CCR5 in the same panel of cell lines. MDA-MB-231 displays slower mobility ($\text{D}=0.14\;\mu {\text{m}}^{2}/\text{s}$) than other cells; cholesterol depletion with MβCD increases D to $0.27\;\mu {\text{m}}^{2}/\text{s}$. Lines indicate the median and the numbers indicate the mean. **d**, Representative PIE-FCCS traces (ACF/CCF) for 2D fits from COS7, HEK293, MDA-MB-231, and MCF-10A cells co-transfected with CXCR4-mGL and CCR5-mS3, highlighting higher amplitude of CCF in MDA-MB-231. Residuals (bottom) show fit deviations. Across-cell-line comparisons were performed using Kruskal-Wallis tests, followed by Dunn’s multiple-comparisons correction; **** and ** denote P < 0.0001 and P < 0.01, respectively. Data pooled from three independent days; ~30–40 cells were analyzed per day per condition. Scale bars, 10μm.

We then compared CXCR4-CCR5 oligomerization across COS7, HEK293, MCF-10A, and MDA-MB-231 cells under identical basal conditions (Fig. 2). Immunofluorescence confirmed plasma membrane expression of both receptors with appropriate isotype controls in all cell lines, and the expected loss of CXCR4 in the KO relative to MDA-MB-231 WT, which shows robust endogenous CXCR4 (Fig. 2a). Under matched expression windows, fractional cross-correlation (fc) differed strongly by cell background; MDA-MB-231 exhibited a significantly higher $f_{c}$ (0.22 ± 0.24) relative to COS7 (0.08 ± 0.19), HEK293 (0.08 ± 0.44), and MCF-10A (0.08 ± 0.18), consistent with a shift toward increased proportion of dimers/trimers/tetramers in the cancer-derived membrane context versus monomer/dimer mixtures in the other cell lines (Fig. 2b; Supplementary Table 1). Diffusion was correspondingly reduced in MDA-MB-231 ($0.14\pm 0.30\;\mu {\text{m}}^{2}\;{\text{s}}^{-1}$) relative to COS7 ($0.22\pm 0.70\;\mu {\text{m}}^{2}\;{\text{s}}^{-1}$), HEK293 ($0.18\pm 0.36\;\mu {\text{m}}^{2}\;{\text{s}}^{-1}$), and MCF-10A ($0.15\pm 0.29\;\mu {\text{m}}^{2}\;{\text{s}}^{-1}$) (Fig. 2c; Supplementary Table 1). Representative ACF/CCF traces (Fig. 2d) show a prominent cross-correlation function amplitude in MDA-MB-231 compared to the other cell lines. Because cholesterol-rich membrane organization is frequently remodeled in invasive breast cancer cells^35^, we asked whether the enhanced CXCR4-CCR5 co-assembly in MDA-MB-231 depends on cholesterol. Prior work reports higher surface cholesterol exposure in malignant breast-cell models compared with non-malignant MCF-10A and shows that MDA-MB-231 similarly displays cholesterol-enriched surface domains and cholesterol-dependent phenotypes^35–37^. Moreover, CXCR4 and CCR5 have been proposed to harbor specific cholesterol-interaction motifs that can influence raft partitioning and conformational stability, supporting a mechanistic link between cholesterol engagement and chemokine-receptor assembly^18,30,38^. Motivated by this literature and by the large basal heteromer signal in MDA-MB-231, we performed acute cholesterol depletion in MDA-MB-231 using methyl-β-cyclodextrin (MβCD; 5 mM). Cholesterol depletion selectively reduced fc (to 0.07 ± 0.12) and increased diffusion (to $0.27\pm 0.47\;\mu {\text{m}}^{2}\;{\text{s}}^{-1}$), consistent with cholesterol-dependent stabilization of CXCR4-CCR5 assemblies in this membrane context (Fig. 2b,c). Together, these data establish that CXCR4-CCR5 heteromerization is strongly cell dependent, with cancer-derived membranes favoring assembly relative to the representative non-cancer model cell lines examined here.

### Ligands bias CXCR4-CCR5 assembly, trafficking, and signaling dynamics in live cells

**Figure 3.**
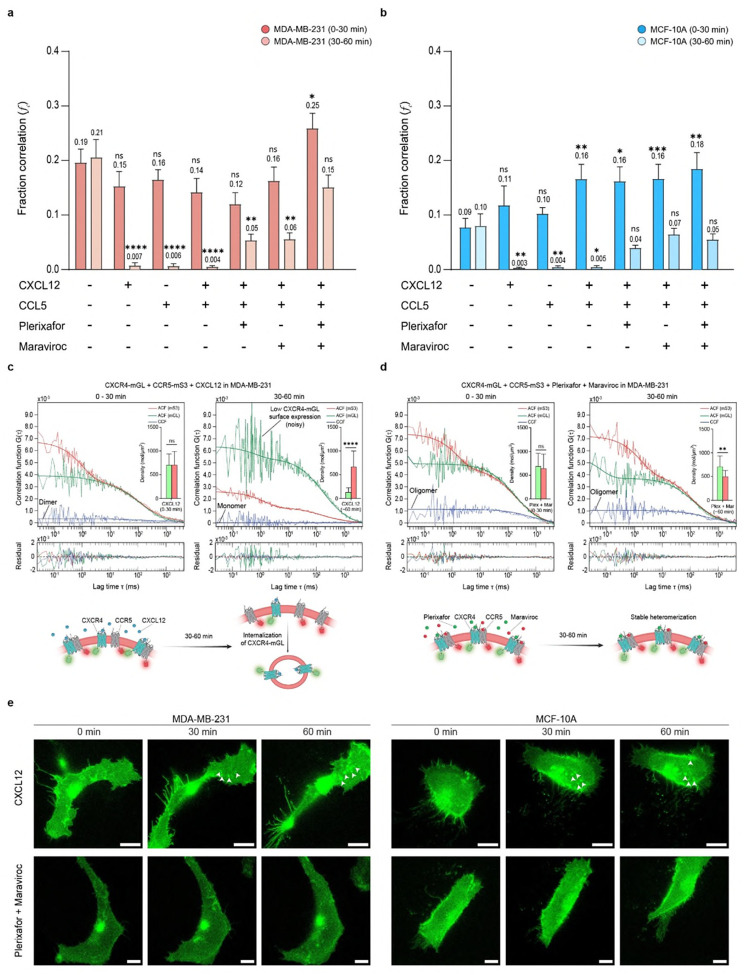
Ligand- and time-dependent CXCR4-CCR5 heteromerization in MDA-MB-231 and MCF-10A cells. **a,b**, Fractional cross-correlation ($f_{c}$) between CXCR4-mGL and CCR5-mS3 measured by PIE-FCCS in MDA-MB-231 (CXCR4-KO; a) and MCF-10A (b) cells under the indicated ligand conditions at two post-treatment windows; 0–30 min and 30–60 min. Agonists (CXCL12, CCL5, or CXCL12+CCL5; 100 nM) remodel CXCR4-CCR5 co-assembly over time, with fc values decreasing toward 30–60 min, whereas antagonists (plerixafor, maraviroc, or plerixafor+maraviroc; 1000 nM) maintain elevated fc over the same interval. Bar plots show mean ± SEM and values above bars indicate means. Statistical significance was assessed by Kruskal-Wallis tests followed by Dunn’s multiple-comparisons correction, comparing each time window (0–30 min and ~60 min) to the basal condition within each cell line. **c**,**d**, Representative PIE-FCCS autocorrelation and cross-correlation functions (ACF/CCF; 2D fits) from MDA-MB-231 cells treated with CXCL12 (**b**) or plerixafor + maraviroc (**c**) at 0–30 min and 30–60 min. CXCL12 treated cells show reduced surface CXCR4-mGL signal by 30–60 min, consistent with internalization and reflected by increased noise in the green-channel ACF, whereas antagonist-treated cells retain comparatively stable surface expression. Residuals (bottom) indicate fit deviations. Statistical comparisons in **c**,**d** were performed using unpaired two-tailed t-tests. **e**, Representative time-lapse live-cell imaging of MDA-MB-231 (CXCR4-KO) and MCF-10A co-expressing CXCR4-mGL and CCR5-mS3 following CXCL12 or plerixafor+maraviroc over 60 min (Supplementary Movie 1). Arrowheads mark puncta consistent with CXCR4 internalization. ns, not significant; *, P < 0.05; ** < 0.01; *** < 0.001; ****; P < 0.0001. Data pooled from three independent days; ~30–40 cells analyzed per condition per day. Scale bars, 10μm.

We next asked whether ligands modulate CXCR4-CCR5 oligomerization at the plasma membrane. In MDA-MB-231 and MCF-10A, agonists (CXCL12, CCL5, or both; 100 nM) produced a time-dependent remodeling of CXCR4-CCR5 assemblies accompanied by modest changes in diffusion, consistent with ligand-driven trafficking (Fig. 3a,b; Supplementary Tables 4,5). For instance, in MDA-MB-231 where basal heteromerization was high (fc=0.19±0.18), CXCL12 reduced fc to 0.15 ± 0.14 within 0–30 min and further to 0.07 ± 0.05 by 30–60 min, with the late time point significantly lower than the early window (Fig. 3a). This decrease was accompanied by a marked drop in the green channel surface density (noisy green ACF) from 645.23 ± 301.66 to $229.19\pm 274.07\;\text{mol}/\mu {\text{m}}^{2}$, consistent with depletion of surface CXCR4-mGL via internalization (Fig. 3c; Supplementary Table 4). In contrast, antagonists (plerixafor, maraviroc, or both; 1000 nM) produced sustained cross-correlation behavior over the same interval, with $f_{c}$ values that were statistically indistinguishable between 0–30 min and 30–60 min in most conditions (Fig. 3a) and with comparatively stable diffusion profiles (Supplementary Tables 4,5). For example, the plerixafor + maraviroc combination in MDA-MB-231 yielded $f_{c}$ value = 0.25 ± 0.05 at 0–30 min that remained relatively high at 30–60 min (fc=0.15±0.06), with essentially unchanged surface density ($518.78\pm 277.36\;\text{mol}/\mu {\text{m}}^{2}$) over the same period (Fig. 3d; Supplementary Table 4). Time-lapse imaging captured the trafficking outcome; agonist exposure induced intracellular puncta consistent with ligand-triggered receptor internalization in both MDA-MB-231 and MCF-10A, whereas antagonist treatment maintained predominantly surface-localized fluorescence with no puncta formation over 60 min (Fig. 3e; Supplementary Movie 1).

These ligand effects were reproducible in MDA-MB-231 and MCF-10A but dampened in COS7 and HEK293 cells. In these lines, antagonists produced lower-amplitude, less stable cross-correlation changes, and comparatively larger diffusion shifts relative to MDA-MB-231/MCF-10A (Supplementary Fig. 5a,b; Supplementary Table 2,3). Live-cell imaging similarly revealed agonist-dependent puncta consistent with internalization, while antagonists did not elicit detectable endocytic accumulation within 60 min (Supplementary Fig. 5c; Supplementary Movie 2). Collectively, these results establish that ligand class dictates both the stability and endocytosis of CXCR4-CCR5 assemblies; agonists promote transient heteromers complexes that proceed to internalization, whereas antagonists, particularly plerixafor **+** maraviroc, stabilize surface heteromers and suppress endocytic entry, with the strongest effects in MDA-MB-231 and MCF-10A.

Because receptor organization measured by PIE-FCCS could, in principle, be altered by experimental conditions or receptor overexpression, we next tested whether CXCR4 and CCR5 remained capable of canonical Gαi signaling under the same conditions. To test whether CXCR4-CCR5 assemblies engage canonical Gαi signaling under our imaging conditions, we measured forskolin-stimulated cAMP accumulation in MDA-MB-231 and MCF-10A cells expressing CXCR4-CCR5 (Supplementary Fig. 6a). Forskolin alone produced a robust elevation of cAMP in both lines relative to vehicle (DMSO). Application of CXCL12 or CCL5 (100 nM) significantly reduced the forskolin response, and the combination of both chemokines produced a comparable suppression, consistent with Gαi-mediated inhibition of adenylyl cyclase. The magnitude of chemokine-induced cAMP reduction was slightly greater in MDA-MB-231 than in MCF-10A, mirroring the enhanced heteromerization seen by PIE-FCCS. CXCR4 and CCR5 antagonists (plerixafor and maraviroc, 1μM) largely reversed the CXCL12/CCL5-dependent inhibition when applied individually and restored cAMP to near-forskolin levels when combined, indicating that the effect arises from specific chemokine engagement of the CXCR4-CCR5 pair rather than off-target signaling. Pertussis toxin (PTX) abolished the ligand-dependent decrease in cAMP, confirming coupling through Gαi. The accompanying structural cartoon (Supplementary Fig. 6b) summarizes this mechanism, CXCL12/CCL5 binding to CXCR4-CCR5 promotes Gαi activation and inhibits adenylyl cyclase, thereby dampening the forskolin-evoked cAMP signal. The slightly stronger cAMP suppression in MDA-MB-231 cells parallels the enhanced heteromerization measured by PIE-FCCS, which connects receptor assembly to functional signaling in cancer cell membranes.

### Membrane cholesterol composition dictates CXCR4-CCR5 interface and heteromer stability

**Figure 4.**
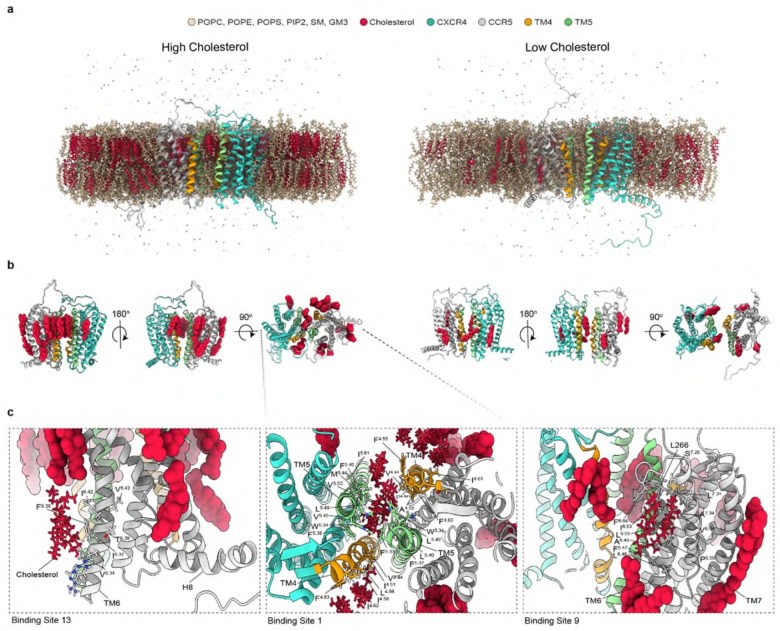
Binding modes of CXCR4-CCR5 dimers in membranes mimicking MDA-MB-231 and MCF-10A cells. **a**, CXCR4-CCR5 dimers embedded in all-atom bilayer models representative of MDA-MB-231- and MCF-10A-like plasma membrane. CXCR4 is shown in turquoise, CCR5 in grey, TM4 in orange, TM5 in green, cholesterol in red, and bulk lipids (POPC, POPE, POPS, PIP2, sphingomyelin, GM3) in tan. **b**, Cholesterol distribution at the CXCR4-CCR5 interface in MDA-MB-231- and MCF-10A-like systems from different viewing angles, highlighting cholesterol molecules (red spheres) belonging to the highest-scoring binding sites identified by PyLipID. **c**, CXCR4-CCR5 interface in the MDA-MB-231-like membrane showing binding sites 1, 9, and 13, which exhibit the highest cholesterol residence scores. Superscripts correspond to Ballesteros-Weinstein numbering.

To test whether the enhanced heteromer stability observed by PIE-FCCS in MDA-MB-231 cells can be explained by cholesterol-dependent receptor interfaces, we performed complementary coarse-grained (CG) and all-atom (AA) simulations of CXCR4-CCR5 embedded in asymmetric bilayers that mimic high and low cholesterol lipidomes (Fig. 4a). Across force-fields and resolutions, high-cholesterol membranes (MDA-MB-231-like) favored compact, long-lived dimers, whereas lower-cholesterol membranes (MCF-10A-like) sampled weaker, short-lived contacts (Fig. 4a; Supplementary Movie 3). In high-cholesterol bilayers, AA simulations recovered a dominant interface family centered on the lateral TM4/TM5 face, with steadily increasing H-bond counts (Supplementary Fig. 7a), reduced protomer center-of-mass (COM) separations (Supplementary Fig. 7b), increasing buried surface area (ΔSASA) over time (Supplementary Fig. 7c), and lower interfacial RMSD/RMSF (Supplementary Fig. 7d-f). By contrast, low-cholesterol membranes exhibited shorter H-bond lifetimes, broader COM distributions, smaller buried surfaces (higher ΔSASA), and elevated interfacial RMSD/RMSF, consistent with partially engaged, transient states (Supplementary Fig. 7a-f).

**Figure 5.**
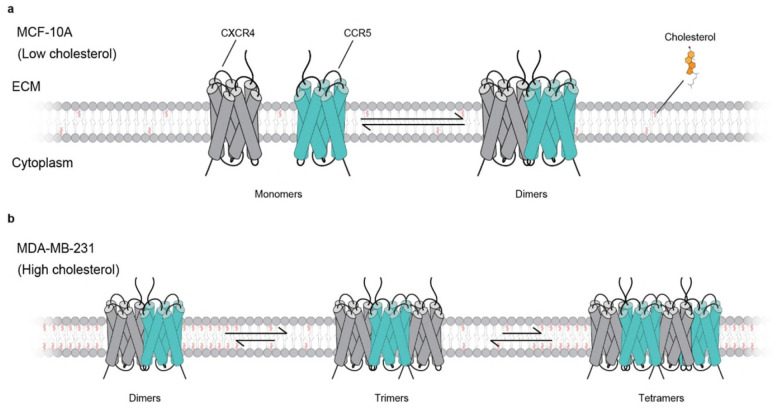
Conceptual model of CXCR4-CCR5 organization in MDA-MB-231 versus MCF-10A membranes. **a**, In MCF-10A-like, low-cholesterol membranes, CXCR4-CCR5 predominantly samples a monomer-dimer equilibrium. **b**, In MDA-MB-231-like, cholesterol-enriched membranes, CXCR4-CCR5 populates higher-order assemblies (dimers, trimers, tetramers), most frequently via a symmetric TM4/TM5 interface, but potentially also through asymmetric contacts involving TM6/TM7, TM1, or TM5/TM6/TM7 of CXCR4 and TM1/TM2/H8, TM4/TM5, or TM1/H8 of CCR5.

Direct cholesterol engagement was a key determinant of these differences. PyLipID analyses revealed recurrent cholesterol hotspots flanking the dimer seam in high-cholesterol membranes. Residues with high-occupancy and long-residence times formed an annular belt that bridged helices across the interface, with asymmetric lipid contacts suggesting stronger anchoring on the CCR5 side (Fig. 4b; Supplementary Figs. 8,9). These sites were fewer and less persistent in low-cholesterol bilayers. Among the most stabilizing pockets (sites 1, 9, 13) in high-cholesterol bilayers, interactions were predominantly hydrophobic (Fig. 4c). For site 1, CXCR4 TM4/TM5 residues V155^4.44^, I162^4.51^, L167^4.56^, I169^4.58^, I173^4.62^, F174^4.63^, W195^5.34^, F199^5.38^, F201^5.40^, M205^5.44^, V206^5.45^, L210^5.49^, V214^5.53^, and I221^5.60^ contributed to the interaction alongside CCR5 TM4/TM5 residues V147^4.44^, F158^4.55^, A159^4.56^, P162^4.59^, I164^4.61^, F166^4.63^; F189^5.33^, W190^5.34^, F193^5.37^, L196^5.40^, and L205^5.49^. For site 9, CCR5 TM6 residues L246^6.46^, F247^6.47^, A249^6.49^, P250^6.50^, I253^6.53^, V254^6.54^, L255^6.55^, F264^6.64^, and TM7 residues S270^7.26^, L275^7.31^, A278^7.34^, and ECL3 residue L266 engaged cholesterol. For site 13, CCR5 TM6 residues V234^6.34^, I237^6.37^, F238^6.38^, T239^6.39^, I242^6.42^, and V243^6.43^ remained cholesterol-bound throughout the run (superscripts correspond to Ballesteros-Weinstein numbering). Frames with high cholesterol residence coincided with more compact interfaces (lower ΔSASA and COM), whereas reduced cholesterol engagement aligned with solvent-exposed conformers. Hydrogen bonding further stabilized the interactions in high-cholesterol membranes mainly through four persistent polar residues spanning the heteromer interface: N-terminus (CCR5 S6-CXCR4 D10, CCR5 Q4-CXCR4 T13), ECL2 (CCR5 Q186-CXCR4 N192), ICL2 (CCR5 F135^34.52^-CXCR4 N143^34.52^), and TM5-TM4 (CCR5 S215^5.59^-CXCR4 K154^4.43^) (Supplementary Fig. 10). These simulations support a cholesterol-dependent coupling between local cholesterol binding and the geometry/lifetime of the CXCR4-CCR5 dimer, providing a structural basis for our live-cell observations. High cholesterol membranes favor stable, higher-order heteromers, whereas low cholesterol membranes bias the ensemble toward transient, weakly engaged dimers (Fig. 5a,b). Together, these simulations provide a structural mechanism for the PIE-FCCS observations, showing that cholesterol stabilizes dimerization interfaces.

## Discussion

Here, we establish a calibrated PIE-FCCS workflow that quantifies GPCR co-assembly, mobility, and surface abundance in living cell membranes, and use it to interrogate class A GPCR dimerization in diverse cell contexts. By resolving co-diffusion rather than proximity, PIE-FCCS quantifies heteromerization, diffusion, molecular brightness, and receptor surface density from a single acquisition, while pulsed interleaving suppresses spectral bleed-through and improves cross-correlation accuracy.^39^ Notably, because PIE-FCCS operates well at modest expression levels, it can resolve GPCR dimerization in regimes where smFRET typically loses sensitivity, an advantage that is critical for class A GPCRs whose assemblies are often sparse, transient, and density-dependent^40^.

The approach is not without limitations. It preferentially samples mobile species, such that immobile or sub-diffraction nanodomains contribute weakly to the fluctuation signal; fluorescent fusion tags, expression levels, and fluorophore photophysics can influence apparent brightness and diffusion. Furthermore, while PIE-FCCS constrains when and to what extent receptors co-diffuse, it does not resolve atomic details of receptor packing, and interface geometry therefore requires structural and multiscale modeling, ideally validated in native membranes.

Prior evidence for CXCR4-CCR5 proximity, based on colocalization, BiFC, FRET/BRET, and co-immunoprecipitation, was typically obtained in a single cell background, often at non-physiological expression, and with limited ligand sampling^23–29^. n contrast, live-cell measurements at near-native receptor densities reveal a pronounced cell-type dependence in receptor assembly.

Cancer-derived MDA-MB-231 cells favor stable, higher-order CXCR4-CCR5 mixtures (dimers/trimers/tetramers), whereas COS7, HEK293 and MCF-10A exhibit weaker, more transient assemblies (monomers/dimers). These findings are consistent with the broader view that GPCR quaternary structure is context dependent^7^. Ligand class further modulates heteromer fate. Agonists (CXCL12, CCL5) elicit a rapid, transient increase in CXCR4-CCR5 co-diffusion that diminishes over ~60 min and coincides with receptor internalization, whereas antagonists (plerixafor, maraviroc), especially in combination, stabilize surface complexes, yielding sustained cross-correlation with minimal endocytosis.

Prior BiFC and BRET work reported that chemokine binding remodels pre-formed CXCR4-CCR5 heterodimers and that antagonism of one partner can cross-inhibit the other^26^, consistent with our observation that ligand identity governs complex stability. In immune cells, CD4/CXCR4/CCR5 higher-order assemblies alter HIV-1 envelope binding and downstream cytoskeletal responses^28^, demonstrating that heteromer-specific conformations encode distinct signaling and trafficking outcomes. Consistent with this organizational picture, cAMP assays under the same ligand conditions showed that CXCL12 and CCL5 suppress forskolin-stimulated cAMP in both MDA-MB-231 and MCF-10A, whereas plerixafor and maraviroc, particularly in combination, restore cAMP toward forskolin levels in a pertussis toxin-sensitive manner. These measurements show that CXCR4-CCR5 assemblies couple to Gαi and that ligand-dependent reorganization resolved by PIE-FCCS has direct consequences for downstream signaling. Together, these results support a model in which cell state and ligand bias jointly tune receptor organization, signaling, and trafficking, providing a mechanistic basis for context-dependent GPCR oligomerization.

Membrane composition likely drives these observed differences. Cholesterol and raft lipids are established regulators of membrane receptor conformation and oligomerization^41^, with marked remodeling observed in cancer cells^36^. Consistent with this, computational studies predict cholesterol-dependent CXCR4-CCR5 interfaces whose populations shift with cholesterol content^18,30,38^. Acute cholesterol depletion in MDA-MB-231 cells destabilized the heteromer and increased lateral mobility, in line with classic observations that raft disruption alters membrane protein organization^42^. In our MD analyses, we focused on a low-energy symmetric TM4/TM5 interface, although alternative interfaces are also plausible^18,30^. This multiplicity of interaction modes provides a structural basis for higher-order oligomerization, consistent with the trimers and tetramers observed by PIE-FCCS in MDA-MB-231 cells. Together, these results support a model in which lipid composition shapes the heteromeric landscape, biasing CXCR4-CCR5 toward stable, higher-order assemblies in cholesterol-rich membranes and transient, dimeric states in cholesterol-poor contexts (Fig. 5).

These findings have direct pharmacological implications. Heteromerization can reshape ligand affinity, efficacy, signaling bias and trafficking, creating drug-addressable states distinct from either protomer. The observation that antagonists stabilize CXCR4-CCR5 at the surface suggests opportunities for bivalent ligand designs or rational combination strategies that lock heteromers into non-internalizing, signaling-defined configurations, potentially improving selectivity and durability while reducing off-target effects. These findings align with growing evidence that GPCR heteromers are therapeutically relevant and reinforce the translational value of mapping cell-specific heteromers to anticipate efficacy or resistance across tissues and disease states.

Future work should be done to further define how membrane lipid composition shapes receptor structure and function. For example, quantitative lipidomics and targeted lipid perturbations (e.g., CRISPR-based modulation of lipid enzymes or orthogonal raft modulators) would help establish causal links between cholesterol and heteromer stability. Integrative approaches combining PIE-FCCS with CG/AA simulations and interface-disrupting mutagenesis can refine structural models and identify key contact residues and lipid interactions. Functionally, pairing PIE-FCCS with biosensor readouts (e.g., mini-G, β-arrestin, ERK reporters) would relate heteromer lifetimes to coupling bias and downstream signaling. Moving toward endogenous tagging and primary cell models will further test generality and reduce overexpression artifacts.

Overall, these findings show that GPCR organization is governed by cellular context and ligand class, with membrane composition being a major contributor to receptor assembly and downstream signaling. Together with complementary structural and pharmacological analyses, these findings support heteromer-focused strategies for mechanistic studies and GPCR-targeted therapeutic development.

## Methods

### Plasmid construction

All constructs used the mammalian backbone pcDNA3.1(−) (Invitrogen, Carlsbad, CA, USA; cat. no. V790–20). All protein sequences were retrieved from UniProt database^43^. To generate mGluR2-mGreenLantern and mGluR2-mScarlet3, we first inserted a Kozak-signal peptide-flexible linker cassette between the NheI/XhoI sites of pcDNA3.1(−). The Kozak element comprised GCCACC at −6 to −1 and a G at +4 relative to the start codon (ATG). As a signal peptide, we used the N-terminal 22 aa of mGluR5 (UniProt: P41594-1)^40^, followed by a gly/ser-rich flexible linker (GSAGSAAGSGEF)^44^. The mGluR2 coding region (aa 20–872, UniProt: Q14416) was synthesized and cloned downstream of the mGluR5 signal peptide via BlpI. Fluorescent proteins mGreenLantern (Addgene, plasmid #161912; pcDNA3.1-mGreenLantern, Gregory Petsko)^45^ and mScarlet3 (Addgene, plasmid #189772; pER-mScarlet3_N1, Dorus Gadella)^46^ were then subcloned, each with a stop codon, into the XhoI site 3′ of mGluR2 to yield two constructs of the form: Kozak-signal peptide-mGluR2-flexible linker-FP-stop. Three additional receptors, Δ2Δ (aa 557–857, UniProt: Q14416), CXCR4 (full-length; UniProt: P61073-1), and CCR5 (full-length; UniProt: P51681) were synthesized and swapped in for mGluR2 using NheI/BlpI, producing Δ2Δ-mGreenLantern_N1, Δ2Δ-mScarlet3_N1, CXCR4-mGreenLantern_N1, CXCR4-mScarlet3_N1, CCR5-mGreenLantern_N1, and CCR5-mScarlet3_N1. To build 1xFRB-mGreenLantern_N1 and 1xFKBP-mScarlet3_N1^47^, we replaced the mGluR5 signal peptide + mGluR2 region with the first 11 aa of human Lyn kinase (UniProt: P07948-1)^48^ followed by GP residues (multi-restriction-site spacer) via NheI/BlpI. FRB (aa 2021-2113; UniProt: P42345) and FKBP (aa 2-108; UniProt: P62942) were then synthesized and inserted using ApaI into the mGreenLantern_N1 and mScarlet3_N1 backbones, respectively. Tandem FRB arrays (2xFRB, 3xFRB) were created by cloning into BlpI of the 1xFRB-mGreenLantern_N1 construct, with FRB repeats joined by a Ser-Arg dipeptide linker; 2xFKBP and 3xFKBP were analogously inserted into 1xFKBP-mScarlet3**_**N1 at BlpI using the same Ser-Arg linker between repeats. Signal peptide functionality was assessed with the SignalP-6.0 server (DTU Health Tech, Kgs. Lyngby, Denmark)^49^, and subcellular localization was predicted using DeepLoc-2.1 server (DTU Health Tech, Kgs. Lyngby, Denmark)^50^. Construct synthesis and cloning were performed by GenScript (Piscataway, NJ, USA; Supplementary Fig. 11a-d). All inserts were verified by Sanger sequencing performed by Azenta Life Sciences (South Plainfield, NJ, USA).

### Mammalian cell culture

Wild-type COS7 (ATCC CRL-1651), HEK293 (ATCC CRL-1573), MDA-MB-231 (ATCC HTB-26), and MCF-10A (ATCC CRL-10317) cells were obtained from ATCC (Manassas, VA, USA). COS7 and HEK293 were cultured in DMEM (Corning, NY, USA; cat. no. 10-013-CV) supplemented with 10% (v/v) FBS (Corning, NY, USA; cat. no. 35-010-CV), 1% (v/v) L-glutamine (Corning, NY, USA; cat. no. 25-005-CI), and 1% (v/v) penicillin-streptomycin (Corning, NY, USA; cat. no. 30-002-CI). MDA-MB-231 cells were maintained in high-glucose DMEM (ATCC, Manassas, VA, USA; cat. no. 30–2002) with the same supplements. MCF-10A cells were grown in MECBM (ATCC, Manassas, VA, USA; cat. no. PCS-600-030) supplemented with the MEC growth kit (ATCC, Manassas, VA, USA; cat. no. PCS-600-040) and 1% penicillin-streptomycin (Corning, NY, USA; cat. no. 30-002-CI). Cells were expanded on 100-mm dishes (VWR Avantor, Radnor, PA, USA; cat. no. 10062-880). At ~90% confluence, cultures were washed 2-3 times with 1×PBS, sterile (Corning, NY, USA; cat. no. 21-040-CVR), detached with TrypLE (Gibco, Thermo Fisher Scientific, Waltham, MA, USA, cat. no. 12604013), pelleted, resuspended in fresh medium, and plated into glass-bottom 96-well plates, #1.5H coverslip (Cellvis, Mountain View, CA, USA; cat no. P96-1.5H-N) at ~2-3 × 10^4^ cells per well one day before transfection. For HEK293, MDA-MB-231, and MCF-10A, plates were pre-coated with fibronectin ($20\;\mu \text{g}\;{\text{mL}}^{-1}$; Sigma-Aldrich, St. Louis, MO, USA; cat. no. FC010-5MG) to promote adhesion. All cultures were maintained at 37 °C, 5% CO_2_. Cells were routinely tested mycoplasma-negative using a PCR kit (abm, Richmond, BC, Canada; cat. no. G239). When contamination was detected, cultures were treated with MycoAway (abm, Richmond, BC, Canada; cat. no. G398; Supplementary Fig. 11e). Experiments used cells between passages 5 and 20.

### Generation of *CXCR4*-knockout in MDA-MB-231 cells

The CXCR4-KO in MDA-MB-231 workflow has been described previously^31^. Briefly, we edited the CXCR4 gene in WT MDA-MB-231 cells using a pool of ssODNs and CRISPR-Cas9 RNPs. Asymmetric ssODNs carrying an in-frame premature stop codon were synthesized with phosphorothioate bonds near both 5′ and 3′ ends. Single-stranded DNA oligos CXCR4.1-sso was designed against the sense strand to promote gene knockout (AAG > TTG) edit. In vitro-transcribed (IVT) gRNAs were generated with the Precision gRNA Synthesis Kit (Invitrogen, Thermo Fisher Scientific, Waltham, MA, USA; cat. no. A29377) from PCR-amplified DNA templates and purified with the MEGAclear kit (Invitrogen, Thermo Fisher Scientific, Waltham, MA, USA; cat. no. AM1908). Synthesized gRNA sequences were as follows: CXCR4.1(GAAGCATGACGGACAAGTAC), CXCR4.2 (ACGGCATCAACTGCCCAGAA), and CXCR4.3 (GTAGCGGTCCAGACTGATGA). The ssODNs were co-delivered with Cas9 protein and IVT gRNA as an RNP complex. For each Neon electroporation (Invitrogen, Thermo Fisher Scientific, Waltham, MA, USA; cat. no. MPK5000), 1.5 × 10^5^ cells were mixed with 250 ng IVT gRNA, 1μg TrueCut Cas9 Protein v2 (Invitrogen, Thermo Fisher Scientific, Waltham, MA, USA; cat. no. A36498), and 1μL10μM ssODN, and pulsed at 1,400 V, 10 ms, 4 pulses. Four replicate transfections were plated into a 6-well plate containing 2 mL pre-warmed DMEM per well. At 72 h post-transfection, cells were harvested for next-generation sequencing to confirm editing. Single colonies were identified with the CloneSelect Imager (Molecular Devices, San Jose, CA, USA; cat. no. 403258), expanded, and quality-controlled by Sanger sequencing and flow cytometry to verify functional loss of CXCR4. The CXCR4 knockout in MDA-MB-231 cells was generated by ThermoFisher Scientific (Madison, WI, USA; Supplementary Fig. 4a).

### Mammalian cells transfection and ligands preparation

COS7, HEK293, MDA-MB-231, and MCF-10A cells were seeded into glass-bottom 96-well plates, #1.5H coverslip (Cellvis, Mountain View, CA, USA; cat no. P96-1.5H-N) at ~2–3 × 10^4^ cells per well. At ~60–70% confluence, transfection was performed with Lipofectamine 3000 (Invitrogen, Thermo Fisher Scientific, Waltham, MA, USA; cat. no. L3000015) per the manufacturer’s instructions. Briefly, two 1.5-mL tubes each received 80μL Opti-MEM (Gibco, Thermo Fisher Scientific, Waltham, MA, USA; cat. no. 11058021); to one tube we added 0.25μg plasmid DNA plus 1μL P3000 reagent, and to the other 1μL Lipofectamine 3000. Contents were combined, mixed, incubated 15 min at room temperature, and 20μL of the DNA-lipid complex was dispensed dropwise per well. Cells were then incubated at 37 °C for 18–24 h. The following day, medium was replaced with Opti-MEM for a 2-h starvation prior to data acquisition. CXCL12 (R&D Systems, Minneapolis, MN, USA; cat. no. 350-NS-010) and CCL5 (R&D Systems, Minneapolis, MN, USA; cat. no. 278-RN-010) were reconstituted at 1,250 nM in sterile 1×PBS (Corning, 21-040-CVR) with 0.1% BSA (VWR Avantor, Radnor, PA, USA; cat. no. 10791–790), diluted to 1,000 nM, and aliquoted (20μL). Adding one aliquot to 200μL Opti-MEM per well yielded 100 nM final. Plerixafor (MedChemExpress, Monmouth Junction, NJ, USA; cat. no. HY-10046) and maraviroc (MedChemExpress, Monmouth Junction, NJ, USA; cat. no. HY-13004) were dissolved in DMSO (G-Biosciences, St. Louis, MO, USA; cat no. 786–1388) at 19.9 mM, serially diluted in 1×PBS to 10,000 nM, and aliquoted (20μL) to achieve 1,000 nM final upon addition to 200μL per well. Aliquots were stored at −20 °C; CXCL12/CCL5 were used within 3 months, plerixafor/maraviroc within 1 month of reconstitution. Rapamycin (Thermo Scientific, Thermo Fisher Scientific, Waltham, MA, USA; cat. no. AAJ62473MF) was prepared at 10 mM in DMSO and stored at −80 °C. On the day of use, fresh working aliquots were made by serial dilution in Opti-MEM to 10,000 nM; adding 2μL to 200μL per well produced 100 nM final. Methyl-β-cyclodextrin (MβCD) (Thermo Scientific, Thermo Fisher Scientific, Waltham, MA, USA; cat. no. J66847.14) was freshly prepared in Opti-MEM at 5 mM, sterile-filtered through 0.22μm Minisart syringe filters (Sartorius, Gottingen, Germany; cat. no. 76474–364), dispensed with 5 mL Luer-Lok syringes (BD, Becton, Franklin Lakes, NJ, USA; cat. no. 309646), applied to cells for 30 min at 37 °C, removed by three PBS washes, and cells were then returned to Opti-MEM for immediate measurements. Forskolin (Sigma-Aldrich, St. Louis, MO, USA; cat. no. 276855–100ML) was prepared as a 25 mM stock by dissolving 10 mg powder in 974 μL DMSO. A 3 mM working stock was generated by mixing 120μL of the 25 mM master stock with 880μL DMSO. For cellular treatments, 2μL of the 3 mM working stock were added to 198μL of cells in induction buffer in each well to achieve a final forskolin concentration of 30μM (200μL total volume). Forskolin stocks were aliquoted, stored at −20 °C, and used within 3 months. The induction buffer consisted of 1× PBS supplemented with the phosphodiesterase inhibitors IBMX (500μM) and Ro 20-1724 (100μM) to prevent cAMP hydrolysis. IBMX (Sigma-Aldrich; cat. no. 17018-100MG) stock was prepared by dissolving 50 mg in 1 mL DMSO to yield a 225 mM solution. Ro 20-1724 (MilliporeSigma, Burlington, MA, USA; cat. no. 557502-100MG) stock was prepared by dissolving 100 mg in 3.59 mL DMSO to obtain a 100 mM solution. To make 40 mL of induction buffer, 89μL of 225 mM IBMX stock and 40μL of 100 mM Ro 20-1724 stock were added to 40 mL 1× PBS, resulting in final concentrations of 500μM IBMX and 100μM Ro 20-1724. Pertussis toxin (PTX; MedChemExpress, Monmouth Junction, NJ, USA; cat. no. HY-112779) was reconstituted by adding 50μL 1× PBS to 50 μg lyophilized toxin to generate a 9.5μM stock. For experiments, 5μL of this stock were diluted into 200μL induction buffer in each well to obtain a final PTX concentration of 0.95 nM. For cAMP standards, a 4μM intermediate solution was prepared by mixing 1 μL of 1 mM cAMP with 250μL induction buffer. In a 96-well plate, 100μL induction buffer were added to wells A2-A12, and 200μL of the 4μM cAMP solution were added to well A1. A twofold serial dilution was then performed by transferring 100μL from A1 to A2, A2 to A3, and so on through A11, yielding standard concentrations of 4000, 2000, 1000, 500, 250, 125, 62.5, 31.25, 15.62, 7.81, and 3.90 nM cAMP; well A12 served as the 0 nM cAMP control.

### Immunofluorescence

Immunofluorescence was performed on endogenous and overexpressed CXCR4/CCR5 in WT COS7, HEK293, MDA-MB-231, CXCR4-KO MDA-MB-231, and MCF-10A. Cells were seeded in 96-well plates, #1.5H coverslip (Cellvis, Mountain View, CA, USA; cat no. P96-1.5H-N) to ~60–70% confluence, transfected with CXCR4-mGL or CCR5-mS3 (0.4μg per well), fixed in 4% paraformaldehyde (Thermo Scientific, Thermo Fisher Scientific, Waltham, MA, USA; cat. no. J19943-K2) and stained with APC anti-human CXCR4 (CD184) antibody, clone 12G5 (5μL of 100μg mL^−1^; BioLegend, San Diego, CA, USA; cat. no. 306510) or APC mouse IgG2a, κ isotype control antibody, clone MOPC-173 (5μL of $100\;\mu \text{g}\;{\text{mL}}^{-1}$; BioLegend, San Diego, CA, USA; cat. no. 400222), Alexa Flour 488 anti-human CCR5 (CD195) antibody, clone J418F1 (10μL of $100\;\mu \text{g}\;{\text{mL}}^{-1}$; BioLegend, San Diego, CA, USA; cat. no. 359104) or Alexa Flour 488 rat IgG2b, κ isotype control antibody, clone RTK4530 (10μL of $100\;\mu \text{g}\;{\text{mL}}^{-1}$; BioLegend, San Diego, CA, USA; cat. no. 400625). Nuclei were counterstained with DAPI ($0.8\;\mu \text{g}\;{\text{mL}}^{-1}$; Invitrogen, Thermo Fisher Scientific, Waltham, MA, USA; cat. no. EN62248). Staining was initiated 24 h after transfection, and imaging was performed immediately thereafter.

### Fluorescence imaging

For live-cell time-lapse and fixed-cell immunofluorescence, imaging was performed in glass-bottom 96-well plates, #1.5H coverslip (Cellvis, Mountain View, CA, USA; cat no. P96-1.5H-N). Widefield epi-fluorescence images were acquired on an inverted Nikon Eclipse Ti microscope (Nikon Instruments, Tokyo, Japan) equipped with an MS-2000 automated XY stage (ASI, Eugene, OR, USA) and a CFI Apo TIRF 100×/1.49 NA oil objective (Nikon Instruments, Tokyo, Japan). Excitation was provided by an X-Cite XYLIS LED light source (Excelitas Technologies, Waltham, MA, USA) and was set to 5% power to limit photobleaching and passed through four filter sets (Nikon Instruments, Tokyo, Japan): DAPI (Ex 350/50 nm, Em 460/50 nm, 400-nm long-pass dichroic, cat. no. 96360), GFP (Ex 470/40 nm, Em 525/50 nm, 495-nm long-pass dichroic, cat. no. 96362), mCherry (Ex 560/40 nm, Em 630/75 nm, 585-nm long-pass dichroic, cat. no. 96365), and Cy5 (Ex 620/60 nm, Em 700/75 nm, 660-nm long-pass dichroic, cat. no. 96324). Images were captured with an Evolve 512 EMCCD (Teledyne Photometrics, Tucson, AZ, USA) using NIS-Elements software, version 6.10 (Nikon, Tokyo, Japan). Time-lapse sequences were acquired every 1 min. Acquisition settings (green/red channels) were 16-bit, no binning; exposure time 70 ms during cell search and 200 ms for final images; camera gain was set to 300. Minimal processing in Fiji (ImageJ) software, version 1.54p (NIH, Bethesda, MD, USA; RRID: SCR_003070)^51^ included brightness/contrast adjustment, Gaussian blur, and adding scale bars. For live-cell work, a multi-well stage incubator (ibidi, Grafelfin, Germany; cat. no. 12150) maintained 37 °C and 5% CO_2_.

### Flow cytometry

MDA-MB-231 cells (WT and KO), either untransfected or transiently expressing CXCR4-mGL, were plated at 5 × 10^5^ cells per well in 6-well plates and grown to ~90% confluence. Cells were rinsed once with 1×PBS (Corning, 21–040-CVR), detached with TrypLE (Gibco, 12604013) for 5 min at 37 °C, washed three times with ice-cold 1×PBS, and fixed for 10 min in 4% paraformaldehyde (Thermo Scientific, J19943-K2). After three additional 1×PBS washes, samples were blocked in 2% BSA (VWR Avantor, 10791–790) for 30 min at 37 °C, washed again, and stained in the dark for 30 min at 37 °C with APC-anti-human CXCR4 (CD184) antibody, clone 12G5 (5μL of $100\;\mu \text{g}\;{\text{mL}}^{-1}$; BioLegend, 306510) or APC-mouse IgG2a, κ isotype control antibody, clone MOPC-173 (5μL of $100\;\mu \text{g}\;{\text{mL}}^{-1}$; BioLegend, 400222). Cells were washed three times and resuspended in ice-cold 1×PBS + 2% BSA for flow cytometry. Data was acquired on a BD Accuri C6 Plus flow cytometer (BD Biosciences, San Jose, CA, USA) using the Accuri C6 Plus software, version 1 (2016) (BD Biosciences, San Jose, CA, USA). APC was excited at 640 nm, collected through a 675/25 nm filter, and recorded on FL4; gates were set using unlabeled controls. Analyses were performed in FlowJo software, version 10.8 (FlowJo, LLC, BD, Ashland, OR, USA).

### 3D DNA and 2D SLB preparation

A dual-labeled 3D DNA standard was synthesized to calibrate the confocal volume. We selected a 40-nt sequence with <50% GC content (5′-ACA AGC TGG AGT ACA ACT ACA ACA GCC ACA ACG TCT ATA T-3′). The 5-isomer TAMRA (5-carboxy-tetramethylrhodamine) was coupled to the 5′ end via a Cu(I)-catalyzed azide-alkyne click reaction, and Alexa Fluor 488 was attached to the 3′ end through an NHS ester. Oligos were purified by HPLC and verified by ESI-MS (Supplementary Fig. 11f). An unlabeled complementary strand was synthesized and annealed in excess per the manufacturer’s protocol. The resulting double-stranded, dual-labeled DNA (TAM-40nt-AF488) was diluted in nuclease-free duplex buffer (IDT, Coralville, IA, USA; cat. no. 11-05-01-12) to a final concentration of 100 nM. DNA synthesis, dye conjugation, and purification were performed by IDT (Coralville, IA, USA). 2D supported lipid bilayers (SLBs) were prepared following Lin *et al*. protocol^52^. Glass coverslips (EMS, Hatfield, PA, USA; cat. no. 72223-01) were cleaned and rendered hydrophilic by a 3-min piranha etch using freshly prepared 3:1 98% H_2_SO_4_ (Millipore Sigma, St. Louis, MO, USA; cat. no. 258105–100ML) : 30% H_2_O_2_ (Millipore Sigma, St. Louis, MO, USA; cat. no. HX0636–1), then rinsed ~10 times with water, and dried under 99.99% N_2_ UHP (Linde Gas & Equipment, Danbury, CT, USA; cat no. NI 5.0UH-T). Small unilamellar vesicles (SUVs) were made from 99.97 mol% DOPC (Avanti Polar Lipids, Alabaster, AL, USA; cat. no. 850375C), 0.01 mol% Texas Red DHPE (Invitrogen, Thermo Fisher Scientific, Waltham, MA, USA; cat. no. T1395MP), and 0.02 mol% Oregon Green 488 DHPE (Invitrogen, Thermo Fisher Scientific, Waltham, MA, USA; cat. no. 012650) dissolved in chloroform (Millipore Sigma, cat. no. CX1058-6). Lipids were dried by rotary evaporation (BUCHI Labortechnik GmbH, Essen, Germany; cat. no. 76320–812) to a thin film, rehydrated in 1 mL 1×PBS to 0.05–2 mg mL^−1^, gently vortexed, and extruded with a lipid extruder (Avanti Polar Lipids, Alabaster, AL, USA; cat. no. 610000) through pre-wetted 10 mm filter supports (Avanti Polar Lipids, Alabaster, AL, USA; cat. no. 610014) and a 100 nm pore size polycarbonate membrane (Avanti Polar Lipids, Alabaster, AL, USA; cat. no. 610005) for ~15 cycles until clear. SUVs were collected, centrifuged (6,000 × g, 10 min), and the supernatant was retained. For bilayer formation, 60−100μL of the SUV suspension was applied to the etched glass for 1–3 min to allow vesicle fusion, then rinsed five times with water (1–5 mL each) and exchanged into 1×PBS for stabilization. Bilayer quality and lateral mobility were verified by FRAP/PIE-FCCS (Supplementary Fig. 1c,d), showing minimal photobleaching and rapid recovery consistent with a continuous, fluid SLB.

### PIE-FCCS optical setup, alignment, and calibration

Pulsed interleaved excitation fluorescence cross-correlation spectroscopy (PIE-FCCS) measurements followed our established configurations. In brief, a custom inverted Nikon Eclipse Ti (Nikon Instruments, Tokyo, Japan) was equipped with a SuperK Extreme pulsed supercontinuum white-laser source, 9.74 MHz (NKT Photonics, Birkerod, Denmark). The output was split with a SpectraK Split tunable splitter (NKT Photonics, Birkerod, Denmark) to generate a 488 ± 10 nm arm and a residual white-light arm, from which 561 nm was isolated using a dichroic mirror, z405/561lrpc (Chroma Technology, Bellows Falls, VT, USA); remaining light was dumped. The 488- and 561-nm lines were cleaned by narrow-band excitation filters, LL01-488-12.5 and LL02-561-12.5 (Semrock/IDEX Health & Science, Rochester, NY, USA) and coupled, respectively, into two single-mode optical fibers, 3 m, QPMJ-3AF3U-488-3.5/125-3AS-18-1-SP and 18 m, QPMJ-3AF3U-488-3.5/125-3AS-3-1-SP (OZ Optics, Ottawa, ON, Canada). The 15 m path-length offset introduced a **~**50 ns inter-pulse delay, enabling pulsed interleaved excitation (PIE) and suppressing spectral cross-talk. Fiber outputs were collimated with infinity-corrected objectives, L-10x (Newport, Irvine, CA, USA) and passed through independently adjustable continuously variable neutral-density (ND) filter wheels, NDC-50C-4M-A (Thorlabs, Newton, NJ, USA). Beams were coaligned into the microscope via a 503 nm single-edge beamsplitter, LM01-503-25 (Semrock/IDEX Health & Science, Rochester, NY, USA) and routed through a custom TIRF cube (91032) housing a dual-band 488/561 excitation filter (zt488/561rpc) and dual-band 488/561 emission filter (zet488/561m) (Chroma Technology, Bellows Fall, VT, USA). Excitation and emission used a CFI Apo TIRF 100×/1.49 NA oil objective (Nikon Instruments, Tokyo, Japan). For time-correlated single-photon detection, emitted light was confocally filtered with a 50μm pinhole, P50K1, and collimated by achromatic lens, AC254-100-A-ML (both from Thorlabs, Newton, NJ, USA), then split by a 560 nm single-edge dichroic beamsplitter, FF560-FDi01-25×36 (Semrock/IDEX Health & Science, Rochester, NY, USA). Green and red channels were defined with 520/44 nm and 612/69 nm emission filters, FF01-520/44-25 and FF01-612/69-25, respectively (Semrock/IDEX Health & Science, Rochester, NY, USA), and focused onto SPAD detectors (Micro Photon Devices, Bolzano, Italy; **~**30 ps timing resolution, $\sim 50\;\mu {\text{m}}^{2}$ active area, ~25 dark counts s^−1^). Single photons were registered on a two-channel TCSPC module, PicoHarp 300 (PicoQuant GmbH, Berlin, Germany) synchronized to the laser reference pulse; acquisition used PicoHarp 300 software, version 3.0 (PicoQuant GmbH, Berlin, Germany).

Alignment and calibration employed a 40-bp dual-labeled DNA (5′ TAMRA / 3′ AF488) to co-register excitation/detection volumes. Excitation powers at the sample were set to 10μW at 488 and 561 nm, as measured with a PM100D power meter (Thorlabs, Newton, NJ, USA). Time-tagged, time-resolved (TTTR) data were collected in 9 × 10 s acquisitions and processed with an in-house MATLAB version R2019a pipeline for PIE-FCCS (MathWorks, Natick, MA, USA). The 3D DNA alignment was performed immediately prior to live-cell PIE-FCCS measurements (Supplementary Fig. 1a,b).

### PIE-FCCS data acquisition and analysis

For live-cell PIE-FCCS, the 488- and 561-nm excitation foci were co-aligned on a flat, peripheral membrane region where the apical-basal spacing is only a few hundred nanometers. The two beams overlapped at the microscope back aperture and focused through the objective to a diffraction-limited spot on the cell edge. This ensured dual-color excitation of mGreenLantern and mScarlet3 tags within the membrane while minimizing cytosolic fluorescence. Typical powers at the sample were set to 0.4μW (488 nm) and 0.8μW (561 nm) using a power energy meter, PM100D (Thorlabs, Newton, NJ, USA) corresponding to $\sim 2.89\;\mu \text{W}/\mu {\text{m}}^{2}$ at 488 nm (green, $\omega_{0}\approx 0.21\;\mu \text{m}$) and $\sim 4.81\;\mu \text{W}/\mu {\text{m}}^{2}$ at 561 nm (red, $\omega_{0}\approx 0.23\;\mu \text{m}$), respectively. Mammalian cells grown on glass-bottom 96-well plates, #1.5H coverslip (Cellvis, Mountain View, CA, USA; cat no. P96-1.5H-N) were maintained at 37 °C in a multi-well stage incubator (ibidi, Grafelfin, Germany; cat. no. 12150). Cells of interest were first identified in widefield epifluorescence mode (GFP and mCherry filter sets) using an Evolve 512 EMCCD camera (Teledyne Photometrics, Tucson, AZ, USA). The PIE excitation spots were then aligned over the chosen membrane region, and time-tagged, time resolved (TTTR) photon streams were acquired with PicoHarp 300 software, version 3.0 (PicoQuant GmbH, Berlin, Germany). For each cell, six 10 s acquisitions were recorded at the same location. Time-gating used TCSPC micro-time (lifetime) windows: gate A (red) 2350–3100 and gate B (green) 1–600 channels, corresponding to macro-time windows of 58.90–77.70 ns and 0.025–15.04 ns, respectively. Intensity traces $F_{i}(t)$ were binned at 10μs temporal resolution. Macro-times within gates A (red) and B (green) produced fluctuation vectors $F_{R}(t)$ and $F_{G}(t)$. Red and green autocorrelation functions $G_{i}(\tau )$ (ACFs; equation 1) were computed from the fluorescence fluctuation traces $F_{R}(t)$ and $F_{G}(t)$. The cross-correlation function $G_{X}(\tau )$ (CCF; equation 2) was then derived from the two $\text{ACFs}\;G_{R}(\tau )$ and $G_{G}(\tau )$. For each cell, six 10 s acquisitions yielded six ACFs/CCFs curves that were subsequently averaged.

(1) $$G_{i}(\tau )=\frac{\left\langle \delta F_{i}(t)\cdot \delta F_{i}(t+\tau )\right\rangle }{{\left\langle \delta F_{i}(t)\right\rangle }^{2}}$$

(2) $$G_{X}(\tau )=\frac{\left\langle \delta F_{R}(t)\cdot \delta F_{G}(t+\tau )\right\rangle }{\left\langle \delta F_{R}(t)\right\rangle \cdot \left\langle \delta F_{G}(t)\right\rangle }$$

(3) $$\delta F_{i}(t)=F_{i}(t)-\left\langle F_{i}(t)\right\rangle$$

Here, $\delta F_{i}(t)$ is the deviation from the temporal mean (equation 3), t is time, τ is the correlation lag time, and the i refers to either the red or green channel. Averaged curves were fit by nonlinear least squares to either a 3D diffusion model for the solution DNA standard (equation 4) or a 2D Brownian model for membrane proteins (equation 5), each including a triplet term.

(4) Gi(τ)=1Ni1−Fi−Fie−ττT,i1−Fi11+ττD,i11+ττD,i+k2

(5) Gi(τ)=1Ni1−Fi−Fie−ττT,i1−Fi11+ττD,i

Where $F_{i}$ denotes the triplet-state fraction with lifetime $\tau_{T,i};\tau_{D,i}$ is the mean dwell time for molecules to move through the observation volume; $\left\langle N_{i}\right\rangle$ is the mean number of diffusing molecules; and κ is the axial-to-lateral aspect ratio of the confocal volume ($k=z_{0}/\omega_{0}$). Zero-lag amplitudes of the correlation function for each channel are related to the brightness and the population of diffusing species. Let species be indexed by s=1…n. The channel-specific molecular brightness is $\varepsilon_{i,s}$ and the mean population is $\left\langle N_{s}\right\rangle$ (channel-independent) as shown in equation (6) and equation (7).

(6) $$G_{i}(0)=\frac{\sum_{s=1}^{n}\varepsilon_{i,s}^{2}\left\langle N_{s}\right\rangle }{{\left(\sum_{s=1}^{n}\varepsilon_{i,s}\left\langle N_{s}\right\rangle \right)}^{2}}\text{for}\;i,j\in \{\text{green},\text{red}\}$$

(7) $$G_{X}(0)=\frac{\sum_{s=1}^{n}\varepsilon_{i,s}\varepsilon_{j,s}\left\langle N_{s}^{\left(ij\right)}\right\rangle }{\left(\sum_{s}\varepsilon_{i,s}\left\langle N_{S}\right\rangle \right)\left(\sum_{s}\varepsilon_{j,s}\left\langle N_{S}\right\rangle \right)},\;i\neq j$$

Only doubly labeled, co-diffusing complexes contribute to $\left\langle N_{s}^{\left(ij\right)}\right\rangle$. If all species have the same brightness in each channel, $G_{G}(0)$ (or $G_{R}(0)$ reduces to $1/\left\langle N_{S}\right\rangle$ for that channel. From the fitted populations and calibrated Gaussian detection volumes, surface density and solution concentration are calculated as shown in equation (8) and equation (9).

(8) $$\sigma =\frac{\left\langle N\right\rangle }{\pi \omega_{0}^{2}}$$

(9) $$C=\frac{\left\langle N\right\rangle }{\pi^{3/2}\omega_{0}^{2}z_{0}}$$

Where σ is the surface density, C is the solution concentration, $\omega_{0}$ is the lateral $1/e^{2}$ intensity radius of a Gaussian detection profile $W(r)\propto /e^{-2r^{2}/\omega_{0}^{2}}$ with effective area $A_{\text{eff}}=\pi \omega_{0}^{2}$ for 2D diffusion, ${\text{z}}_{0}$ is the axial $1/e^{2}$ intensity radius of a Gaussian detection profile $W(r,z)\propto /e^{-2r^{2}/\omega_{0}^{2}}e^{-2z^{2}/{\text{z}}_{0}^{2}}$ with effective volume $V_{\text{eff}}=\pi^{3/2}\omega_{0}^{2}z_{0}$ for 3D diffusion. Using the confocal volumes and the fitted dwell times, 2D and 3D particle diffusion coefficients ($D_{i}$) can be calculated as shown in equation (10).

(10) $$D_{i}=\frac{\omega_{0}^{2}}{4\tau_{D,i}}$$

Although the zero-lag CCF amplitude, $G_{X}(0)$, scales with the number of co-diffusing species, a more robust index of binding is the faction correlated $f_{c}$ value which reports the ratio of co-diffusing population to the limiting constituent (equation 11). After fitting the correlation curves, $f_{\text{c}}$ is computed from the initial amplitudes $G_{G}(0),G_{R}(0)$, and $G_{X}(0)$; by definition $f_{c}\in [0,1]$, spanning no co-diffusion to complete co-diffusion.

(11) $$f_{c}=\text{min}\left(\frac{G_{x}(0)}{G_{R}(0)},\frac{G_{x}(0)}{G_{R}(0)}\right),0\leq f_{c}\leq 1$$

Given the nanosecond timing of detected photons, ACFs/CCFs were computed with a multiple-tau correlator (PAM-PIE Analysis with MATLAB)^53^. All correlation sets were quality-checked, and traces showing artefacts (e.g., detector afterpulsing, non-diffusive noise, or intracellular contamination) were excluded prior to fitting.

### Fluorescence lifetime data analysis

Photon steams form PIE-FCCS were accumulated into fluorescence-lifetime histograms. For each cell, five 10 s acquisitions were averaged, deconvoluted with the instrument response function (IRF), and fit with a single-exponential decay model to obtain the mGreenLantern/AF488 lifetime ($\tau_{1}$) in presence of acceptor. Mean FRET efficiency was then computed as shown in equation (12).

(12) $$E_{\text{FRET}}=100\%\;x\;\left(1-\frac{\tau_{1}}{\tau_{0}}\right)$$

Where $\tau_{0}$ denotes the donor-only lifetime, measured either for CXCR4-mGL in cells expressing mGreenLantern alone or for the AF488-labeled DNA without TAMRA (acceptor).

### Live cell cAMP assay

Agonist-induced Gαi coupling was quantified using the cAMP-Glo^™^ assay (Promega, Madison, WI, USA; cat no. V1501) according to the manufacturer’s instructions with minor modifications. MDA-MB-231 and MCF-10A cells were plated in flat-bottom, white, tissue-culture-treated 96-well plates (Greiner Bio-One, Monroe, NC, USA; cat no. 655083) at ~2–3 × 10^4^ cells per well. At 60–70% confluence, cells were co-transfected with CXCR4-mGL and CCR5-mS3 using Lipofectamine 3000 (Invitrogen, Thermo Fisher Scientific, Waltham, MA, USA; cat no. L3000015) as described above and maintained under the same culture conditions. Twenty-four hours after transfection, cells were serum-starved for 2 h and then incubated in serum-free induction buffer (1× PBS, 500μM isobutyl-1-methylxanthine (IBMX), 100μM Ro 20-1724) containing the indicated treatments: vehicle (DMSO), forskolin (30μM), CXCL12 (100 nM), CCL5 (100 nM), plerixafor (1,000 nM), maraviroc (1,000 nM), or pertussis toxin (PTX; 0.95 nM). Cells were treated with PTX 16–18 hours before ligand treatment. Following a 15-min incubation at 37 °C, 30μL of cAMP-Glo^™^ Lysis Buffer were added to each well (including wells containing cAMP standards), and plates were shaken at room temperature for 15–30 min. Subsequently, 40μL of cAMP Detection Solution (prepared by mixing 2.5μL Protein Kinase A with 1,000μL cAMP-Glo^™^ Reaction Buffer) were dispensed into all wells, mixed by shaking for 30–60 s, and incubated for 20 min at room temperature. Finally, 80μL of Kinase-Glo^®^ Reagent were added, mixed for 30–60 s, and plates were incubated for an additional 10 min before luminescence acquisition on a Synergy H1 multi-mode microplate reader (Agilent BioTek, Winooski, VT). A fresh cAMP standard curve was generated with each experiment. For standards, ΔRLU was defined as the difference in relative light units between the 0 nM cAMP well and each standard concentration; for cell samples, ΔRLU was calculated as the difference between untreated (vehicle) and treated wells. Cellular cAMP concentrations (nM) were interpolated from the standard curve. Each condition was measured in five biological replicates, and data visualization and statistical analyses were performed GraphPad Prism software version 10 (GraphPad, Boston, MA, USA).

### System setup for MD simulations

Amino acid sequences for full-length CXCR4 (UniProt: P61073–1) and CCR5 (UniProt: P51681) were retrieved from UniProt^43^; these receptors were already fused to fluorescent proteins in our expression plasmids for live-cell studies. Structures were predicted with AlphaFold3, then superimposed to experimental templates in ChimeraX version 1.10 (UCSF, San Francisco, CA, USA)^54^, using the MatchMaker tool (Needleman-Wunsch alignment; iteration cutoff 2) to compute RMSDs. For CXCR4, we overlaid AlphaFold models with PDB entries 3ODU, 3OE0, 4RWS, 8U4N, 8U4O, 8U4P, 8K3Z, 8U4Q, 8U4R, 8ZPN, 8ZM, and 8ZPL; for CCR5, with 4MBS, 7F1S, 7F1Q, 7F1R, 7F1T, 7O7F, 5UIW, 6AKX, 6AKY, 6MEO, and 6MET (Supplementary Fig. 12a,b). We selected 8U4N (CXCR4) and 7F1S (CCR5) as working structures based on completeness, resolution, lack of mutations, and low RMSD. Missing segments and any sequence discrepancies were identified by comparing predicted and experimental models in ChimeraX and through multiple sequence alignment in Clustal Omega (EMBL-EBI, Cambridgeshire, UK)^55^ (Supplementary Fig. 12c). Gaps were rebuilt by homology modeling in MODELLER version 10.4^56^ in ChimeraX using the AlphaFold prediction as template. Side-chain replacements and initial clash relief were performed in ChimeraX (Rotamers tool), followed by structure preparation (hydrogens, charges, etc.) with Dock Prep tool. Final checks in ChimeraX verified sterics, rotamer geometry, and hydrogen-bonding networks. Prepared CXCR4 and CCR5 protomers were placed ~4 nm apart in the membrane plane with TM4/5 interfaces facing each other, using ChimeraX for lateral orientation and the PPM version 3.0 server (University of Michigan, Ann Arbor, MI, USA)^57^ for z-axis embedding (Supplementary. 12d). Coarse-grained membrane-protein systems were assembled with CHARMM-GUI Martini Maker (Lehigh University, Bethlehem, PA, USA)^58,59^ using the martini22 force field. Each system comprised a rectangular bilayer (26.2 × 26.2 × 19.4 nm; replacement method) with asymmetric leaflets configured to mimic either cell type^42,60–63^. MDA-MB-231-like system: upper leaflet, 30% POPC / 40% cholesterol / 30% sphingomyelin / 5% glycolipids; and lower leaflet, 10% POPC / 30% cholesterol / 30% POPE / 20% POPS / 15% PIP_2_. MCF-10A-like system: upper leaflet, 40% POPC / 20% cholesterol / 30% sphingomyelin / 5% glycolipids; and lower leaflet, 10% POPC / 20% cholesterol / 30% POPE / 20% POPS / 15% PIP_2_. Both systems were solvated, ionized to 0.15 M NaCl, minimized and equilibrated at 310 K and 1 bar. For all-atom models, the same CXCR4-CCR5 protomers (initially separated by ~4 nm) were embedded with CHARMM-GUI Membrane Builder^64^ using identical lipid asymmetries. Each AA system used a 16.2 × 16.2 × 19.4 nm bilayer, was solvated with 0.15 M KCl, minimized with CHARMM36m^65^ (hydrogen mass repartitioning), and equilibrated under NPT (310 K, 1 bar). Final assemblies were reloaded into ChimeraX for geometry/steric validation prior to use as inputs for MD simulation.

### Coarse-grained (CG) MD simulations

CG simulations were carried out with GROMACS 2024.3^66^ on the Texas Tech University High Performance Computing Center (HPCC), using input systems generated by CHARMM-GUI Martini Maker. Each system consisted of coarse-grained CXCR4-CCR5 protomers embedded in an asymmetric lipid bilayer and solvated with CG water and counterions under periodic boundary conditions. Van der Waals interactions were treated with the potential-shift-Verlet scheme, and long-range electrostatics were modeled with the reaction-field method. Energy minimization was performed in two stages with the steepest-descent algorithm, first using soft-core potentials followed by conventional minimization. During equilibration, the integration time step was ramped from 2 fs to 20 fs while positional restraints on protein and lipid headgroups were progressively relaxed over five consecutive NPT steps. A semi-isotropic Berendsen barostat maintained pressure at 1 bar and a velocity-rescale thermostat held the temperature at 310 K. Production trajectories were then run in the NPT ensemble using a Parrinello-Rahman barostat^67^ with the same non-bonded interaction settings. Energies and coordinates were saved every 100 ps over 30μs of simulation time. CG simulations were initiated with CXCR4-CCR5 protomers separated by 3, 4, 5, 6, 7, and 8 nm, and a 4 nm starting distance was identified as optimal, yielding stable sampling over 30μs of simulation time.

### All-atom (AA) MD simulations

Guided by the CG simulations, we next performed AA MD simulations with CXCR4 and CCR5 protomers initially positioned 4 nm apart. System topologies and starting coordinates were generated using CHARMM-GUI Membrane Builder. All covalent bonds were constrained with the LINCS algorithm^68^, and van der Waals interactions were treated with a force-switching function between 1.0 and 1.2 nm. Long-range electrostatics were computed using the particle-mesh Ewald (PME) method^69^ with a 1.2 nm real-space cutoff, and short-range forces were evaluated with the Verlet cutoff scheme. Both membrane systems were energy-minimized using steepest descent until the maximum force fell below 1000 kJ mol^−1^ nm^−1^, with positional restraints applied to backbone atoms, side chains, and lipid headgroups during this phase. Equilibration followed in six sequential NVT/NPT stages, during which positional restraints on lipid atoms, side chains, and backbone, as well as dihedral restraints, were gradually relaxed to allow full system accommodation. Pressure was maintained at 1 bar using a C-rescale barostat with semi-isotropic coupling, and temperature was held at 310 K with a V-rescale thermostat. Production simulations were then run for 0.5μs in the NPT ensemble using the same barostat and thermostat. For each membrane composition, three independent replicate simulations were conducted. Resulting trajectories were analyzed for center-of-mass (COM) separation, ΔSASA, RMSD, RMSF, hydrophobic contacts, and inter-receptor hydrogen bonds to assess dimer stability under different cholesterol conditions. ΔSASA was computed as shown in equation (13).

(13) $$\Delta \text{SASA}(t)={\text{SASA}}_{\text{complex}}(t)-\left[{\text{SASA}}_{\text{CXCR}4}(t)+{\text{SASA}}_{\text{CCR}5}(t)\right]$$

Hydrogen bonds between CXCR4 and CCR5 were quantified using *gmx hbond*. To identify residues forming persistent hydrogen bonds, 50 snapshots were extracted at 10-ns intervals from the 0.5μs production run and analyzed in ChimeraX.

### Cholesterol-GPCR interaction dynamics

After completion of the production runs, protein-lipid interaction dynamics between cholesterol and the GPCRs were quantified using PyLipID (version 1.5.14)^70^. PyLipID enables automated identification and characterization of lipid-binding sites and residue-level contacts directly from MD trajectories. All cholesterol molecules in the system were included in the analysis. Protein-cholesterol contacts were defined using PyLipID’s dual-cutoff scheme, which distinguishes short-lived fluctuations from bona fide binding and dissociation events; an upper and lower distance cutoff of 0.5 nm were applied for contact detection. Time-resolved lipid-residue contact maps were generated with the LipidInteraction class, from which lipid count, site occupancy, and contact durations were calculated. Normalized survival time correlation functions were fitted with a bi-exponential model to extract residence times and dissociation rate constants. Distinct cholesterol-binding sites were identified by community detection using the Louvain algorithm, and binding-site solvent-accessible surface areas were computed with the Shrake-Rupley method. For each site, we recorded the average contact duration, representative bound poses, residence time, occupancy, and lipid count using PyLipID’s density-based scoring metrics.

### Statistics and reproducibility

Unless noted otherwise in the figure legends, data in the main and supplementary figures are reported as mean ± SEM from at least three independent experiments. PIE-FCCS datasets were processed in MATLAB version R2019a (MathWorks, Natick, MA, USA) and summarized in Excel. Statistical tests, comparisons, and sample sizes are specified in each figure and legend. All analyses were performed in GraphPad Prism software version 10 (GraphPad, Boston, MA, USA). P values were determined using unpaired t-tests under the assumption of a Gaussian or by Kruskal-Wallis analysis followed by Dunn’s multiple-comparisons correction. Significance was denoted as ****P < 0.0001, ***P < 0.001, **P < 0.01, *P < 0.05, and ns (not significant) P ≥ 0.05.

## Supplementary Files

This is a list of supplementary files associated with this preprint. Click to download.

- SupplementaryInformation.docx
- CXCR4CCR5ManuscriptSupplementaryMovies.zip
- CXCR4CCR5ManuscriptSourceData.zip

## Data Availability

Plasmids, engineered cell line (MDA-MB-231 CXCR4-KO), and all custom analysis and simulation codes used in this study are available from the corresponding author upon reasonable request.
